# Emergence of New Delhi Metallo-β-Lactamase 14–Producing *Klebsiella pneumoniae* Sequence Type 147 Clone in Spain and Outbreak in the Canary Islands

**DOI:** 10.3201/eid3201.251504

**Published:** 2026-01

**Authors:** Pablo Aja-Macaya, Tania Blanco-Martín, Cristian Mateo-León, Cristóbal del Rosario-Quintana, Carmen Piña, Enrique de la Cruz-Tabares, Salud Rodríguez-Pallares, Lucía González-Pinto, Alejandro Beceiro, Marina Oviaño, Germán Bou, Diego García-Martínez de Artola, Jorge Arca-Suárez

**Affiliations:** Servicio de Microbiología Clínica and Instituto de Investigación Biomédica A Coruña (INIBIC), Complexo Hospitalario Universitario, A Coruña, Spain (P. Aja-Macaya, T. Blanco-Martín, S. Rodríguez-Pallares, L. González-Pinto, A. Beceiro, M. Oviaño, G. Bou, J. Arca-Suárez); CIBER de Enfermedades Infecciosas (CIBERINFEC), Instituto de Salud Carlos III, Madrid, Spain (T. Blanco-Martín, A. Beceiro, M. Oviaño, G. Bou, J. Arca-Suárez); Hospital Universitario Nuestra Señora de la Candelaria, Santa Cruz de Tenerife, Spain (C. Mateo-León, D. García-Martínez de Artola); Complejo Hospitalario Universitario Insular-Materno Infantil de Gran Canaria, Las Palmas de Gran Canaria, Spain (C. del Rosario-Quintana, C. Piña); Complejo Hospitalario Universitario Insular-Materno Infantil de Gran Canaria, Las Palmas de Gran Canaria (Enrique de la Cruz-Tabares)

**Keywords:** bacteria, Klebsiella pneumoniae, carbapenemase, New Delhi metallo-β-lactamase, NDM-14, ST147, antimicrobial resistance, outbreak, Canary Islands, Spain

## Abstract

The emergence of a high-risk New Delhi metallo-β-lactamase 14–producing *Klebsiella pneumoniae* sequence type 147 clone is of public health concern because of its rapid international spread. We report cross-border emergence and rapid dissemination of that clone in the Canary Islands, Spain, during 2023–2025. We analyzed 30 isolates recovered during 2023 in detail by reviewing clinical and epidemiologic data, conducting whole-genome sequencing to assess clonal relatedness and analyze resistomes, and performing antimicrobial susceptibility testing of novel therapeutic options through reference broth microdilution. The isolates formed a well-defined cluster, with minimal genomic distance and identical resistomes, confirming the local outbreak. Those clones were also closely related to other international New Delhi metallo-β-lactamase 14–producing *K. pneumoniae* sequence type 147 isolates, supporting the ongoing cross-border expansion of that clone. Aztreonam/avibactam was the most active therapeutic option (MICs <0.125 mg/L). Our findings highlight the need for close monitoring to prevent further dissemination of this clone.

The global rise of New Delhi metallo-β-lactamase (NDM)–producing *K. pneumoniae*, highlighted by surveillance programs such as ATLAS, is becoming an alarming concern (*1*). First detected in Europe in patients who had received healthcare in India in 2009 (*2*), NDM-producing strains have since spread widely, particularly among countries in Europe (*3*), and are closely associated with successful *K. pneumoniae* clones, including sequence types (ST) 147, 11, and 15 (*4*). In Spain, the first NDM-1–producing isolate was detected in 2009 in a *K. pneumoniae* isolate from a man from Spain who had received medical attention in India (*5*). Recent nationwide carbapenemase surveillance studies in Spain have reported prevalence rates of <5% for NDM-producing Enterobacterales in most regions but also a gradual spread across different geographic areas (*6*). However, according to recent data from the country’s Network of Laboratories for the Surveillance of Resistant Microorganisms (RedLabRA; https://cnm.isciii.es/redlabra), among 6,756 carbapenemase-producing Enterobacterales (CPE) isolates detected in 2023, a total of 396 (7.9%) were NDM-producing *K. pneumoniae*, making this type the third most prevalent carbapenemase/microorganism association in Spain (*7*).

Among the lineages of *K. pneumoniae* involved in the spread of NDM enzymes, ST147 is likely one of the most prevalent sequence types globally (*8*). That clone first appeared in the early 1990s and became globally disseminated over the next decade, probably catalyzed by successful selection of alterations in the quinolone resistance-determining regions, as well as by acquisition of the plasmid-encoded *bla*_CTX-M-15_ (*9*). NDM-producing *K. pneumoniae* ST147 isolates have been circulating in Spain for at least a decade (*10*). Treatment of infection with those isolates represents a substantial challenge because the strains often carry multiple antimicrobial resistance determinants, including extended-spectrum β-lactamases, aminoglycoside-modifying enzymes, or other carbapenemases, such as *K. pneumoniae* carbapenemase or oxacillinase (OXA) 48–like oxacillinases (*11*,*12*). Moreover, NDM enzymes also display strong catalytic activity against most β-lactams and are increasingly associated with resistance to recently approved or investigational options, such as cefiderocol and cefepime/taniborbactam (*13*,*14*).

A total of 84 NDM carbapenemase variants had been described as of August 28, 2025 (*15*). Whereas most differ from the prototype NDM-1 by only 1 or a few amino acid substitutions, some of those variants have notable effects on β-lactam hydrolysis (e.g., NDM-5) or resistance to newly developed boronate inhibitors (e.g., NDM-9) (*16*,*17*). In 2023, an alarming report described rapid emergence and dissemination of an NDM-14–producing ST147 clone in France, a single NDM-1 amino acid variant (D130G) known to enhance carbapenem-hydrolyzing activity (*18*). The authors identified Morocco as a potential origin and noted high levels of antimicrobial resistance.

Within the framework of the Network of Diagnostic Laboratories for Healthcare-Associated Infection Surveillance in the Canary Islands (*19*), a total of 99 cases of infection with the ST147 clone were identified during January 2023–March 2025 across 3 islands. That outbreak of the NDM-14–producing clone in Spain is among the largest reported in Europe. In line with European Centre for Disease Prevention and Control guidelines for genomic surveillance and multicountry outbreak investigations, we performed a genomic surveillance study, assessed the outbreak’s dimension, and evaluated newly developed therapeutic options to generate timely information for infection control and therapeutic alternatives.

## Methods

### Bacterial Isolates

During January 2023–March 2025, a total of 99 NDM-14–producing *K. pneumoniae* isolates were recovered from clinical or surveillance samples from patients admitted to 4 hospitals on 3 Canary Islands: the Complejo Hospitalario Universitario Insular-Materno Infantil (CHUIMI; Gran Canaria), the Hospital Universitario de Gran Canaria Doctor Negrín (Gran Canaria), the Hospital Universitario Doctor José Molina Orosa (Lanzarote), and the Hospital General de Fuerteventura Virgen de la Peña (Fuerteventura). The isolates were submitted to the reference laboratory of the Laboratory Network for Infection Surveillance System of the Canary Islands (RELAP; Hospital Universitario Nuestra Señora de la Candelaria, Tenerife, Spain). Of the 99 isolates, 30 corresponded to the first nonduplicate NDM-14–producing *K. pneumoniae* isolates identified in the archipelago, all recovered from patients admitted to CHUIMI in 2023. We further selected those 30 isolates for subsequent whole-genome sequence-guided phylogenomic analysis and evaluation of recently approved and investigational β-lactams and β-lactam/β-lactamase inhibitor combinations (Appendix 1 Table 1).

### Clinical and Epidemiologic Data Collection

We screened for carbapenemase production among patients admitted to CHUIMI (Appendix 2) and collected clinical and epidemiologic information related to patients with positive samples for NDM-14–producing *K. pneumoniae*. Researchers anonymized demographic and clinical data with an alphanumeric code and compiled complete details of each sample and the information collected in the initial 2023 outbreak (Appendix 1 Table 1).

### Whole-Genome Sequencing

We sequenced the 30 isolates with an Illumina NovaSeq 6000 (https://www.illumina.com) and obtained total genomic DNA using a Genomic DNA Buffer Set with a Genomic-tip 20/G (QIAGEN, https://www.qiagen.com). We generated Illumina indexed paired-end libraries from a Nextera DNA XT Library Prep Kit (Illumina). In addition, we sequenced the isolate from the index case (I238) identified at CHUIMI, as well as isolate I542 (an NDM-14–producing *K. pneumoniae* isolate that belonged to a phylogenomically distant ST, 997), in parallel with a PacBio platform (Pacific Biosciences, https://www.pacb.com) to obtain high-quality hybrid assemblies and plasmid reconstruction. To understand the evolutionary trajectory, we also sequenced 3 isolates from 2024 (n = 1) and 2025 (n = 2) by using the Illumina NovaSeq 6000.

### Bioinformatic Analysis

We quality controlled reads for bioinformatic assembly with Unicycler version 0.5.0 (https://github.com/rrwick/Unicycler), using a hybrid approach when possible. We then assessed reads for contamination and completeness by using CheckM version 1.1.3 (https://github.com/Ecogenomics/CheckM). We deposited raw reads and assemblies in the National Center for Biotechnology Information under Bioproject PRJNA1216752 and provide complete accession numbers for all databases (Appendix 1 Table 2).

Using *K. pneumoniae* ST147 isolate TGH13 (GenBank accession no. CP012745.1) as a reference, we performed core-genome phylogenomic analysis in snippy version 4.6.0 (https://github.com/tseemann/snippy) with the phylogenetically closest 863 ST147 international isolates from multiple databases (Appendix 1 Table 3). We created a phylogenomic tree by using fasttree version 2.1.11 (https://morgannprice.github.io/fasttree; parameters “-nt -gtr”). We described the complete methodology in detail, including phylogenomic analysis and genomic characterization of isolates (Appendix 2).

### Antimicrobial Drug Susceptibility Testing

We tested for antimicrobial drug susceptibility by using Sensititer EUMDRXXF microdilution plates (Thermo Fisher Scientific, https://www.thermofisher.com), using reference broth microdilution assays with cation-adjusted Müeller-Hinton broth for newly developed β-lactam/β-lactamase inhibitor combinations, and, for cefiderocol, iron-depleted cation-adjusted Müeller-Hinton broth prepared according to Clinical and Laboratory Standards Institute guidelines (*20*). We interpreted MIC values according to EUCAST guidelines (*21*) (Appendix 1 Table 4).

### Molecular Cloning

We cloned the genes *bla*_NDM-1_ and *bla*_NDM-14_ into pUCP-24, then transformed the recombinant plasmids by electroporation into *Escherichia coli* TG1 (wild-type permeability) and *E. coli* HB4 (OmpC and OmpF deficient) and selected on LB agar plates containing 10 mg/L of gentamicin. MICs were determined as described for drug susceptibility (Appendix 1 Table 5).

## Results

### Index Case

The first case of NDM-14–producing *Klebsiella pneumoniae* ST147 (strain I238) in this outbreak was identified on January 13, 2023, in a 34-year-old female patient admitted to CHUIMI. The patient, originally from Laayoune, administered by Morocco, was transferred for urgent intervention after she had a major bile duct injury develop as a complication of a laparoscopic cholecystectomy performed in Morocco in 2022. Bile duct reconstruction surgery was performed on December 28, 2022, and the NDM-14–producing ST147 *K. pneumoniae* isolate was detected 26 days later from the catheter tip and biliary drainage. Targeted intravenous colistin therapy was initiated (4.5 million IU/12 h for 12 days), and the patient was discharged after favorable clinical evolution with continued antimicrobial therapy and outpatient follow-up.

### Epidemiologic Investigation, Outbreak Detection, and Current Extension

The hospital admission of the index case-patient subsequently caused a 3-case transmission event in the digestive surgery unit (ward 10S) at CHUIMI. Control measures were performed, including epidemiologic investigations; systematic screenings of carbapenemase-producing organisms, including culture and molecular-based approaches; enhanced hygiene; transfer to a dedicated high-risk ward (8N); cohorting of patients; and environmental sampling, if needed. Despite control efforts and apparent early containment of cases in ward 10S, RELAP later identified 98 additional NDM-14–producing ST147 *K. pneumoniae* isolates through March 2025, for a total of 99 isolates.

**Figure 1:**
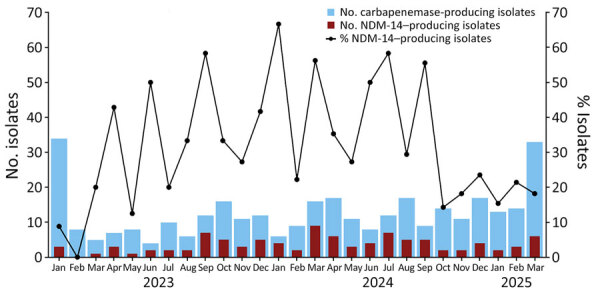
Carbapenemase-producing *Klebsiella pneumoniae* isolates submitted to the Laboratory Network for Infection Surveillance System of the Canary Islands by Complejo Hospitalario Universitario Insular-Materno Infantil (Gran Canaria) during January 2023–March 2025 from study of emergence of NDM-14–producing *K. pneumoniae* sequence type 147 clone in Spain and outbreak in the Canary Islands. Numbers and percentages of NDM-14–producing *K. pneumoniae* isolates compared with all isolates are shown. Isolates were submitted if they fulfilled >1 of the following criteria: recovery from clinical samples associated with invasive infection (excluding colonization); recovery from clinical or environmental sources during outbreak or epidemic events; or detection of emerging pathogens requiring enhanced surveillance, including the NDM-14–producing *K. pneumoniae* isolates described. NDM, New Delhi metallo-β-lactamase.

We compiled a comprehensive representation of the contribution of NDM-14–producing ST147 *K. pneumoniae* to the overall proportion of carbapenemase-producing *K. pneumoniae* isolates submitted by CHUIMI to RELAP during the study period (Figure 1). Isolates that fulfilled >1 of the following criteria were submitted: isolates from clinical samples associated with invasive infection (excluding colonization); isolates from clinical or environmental sources during outbreak or epidemic events; or detection of emerging pathogens requiring enhanced surveillance, including the NDM-14–producing *K. pneumoniae* isolates we describe. The peak of the reported cases occurred during June 2023–September 2024. During that period, the mean (+SD) percentage of submitted *K. pneumoniae* isolates producing NDM-14 among all submitted carbapenemase-producing *K. pneumoniae* isolates was 41.56% (SD +14.40%). The maximum peak of incidence occurred in January 2024, when production of NDM-14 accounted for 67% of the 35 cases.

During the study period, most cases were identified at CHUIMI (n = 93), where the index case was detected. However, during 2024, additional cases were also reported at Hospital Universitario de Gran Canaria Doctor Negrín (n = 4), a hospital in Gran Canaria, the same island as the index case, as well as at Hospital Universitario Doctor José Molina Orosa on the neighboring island of Lanzarote (n = 1) and Hospital General de Fuerteventura Virgen de la Peña on the neighboring island of Fuerteventura (n = 1). Those detections confirmed not only interhospital transmission but also cross-island emergence of NDM-14–producing ST147 *K. pneumoniae*.

To clarify the origin and early epidemiologic dynamics of that clone, we focused on the first 30 cases detected in 2023 at CHUIMI (Appendix 1 Table 1). The isolates were collected from patients across different medical and surgical wards: internal medicine (n = 12); general digestive surgery (n = 4); digestive, hematology, neurology, and neurosurgery (n = 2 each); and pneumology, endocrinology, emergency, vascular surgery, oncology, and infectious diseases (n = 1 each) (Appendix 1 Table 1). The spread of the clone among those wards and departments occurred despite implementation of control measures and could not be clearly linked or assigned to a specific reservoir or transmission chain on which to act, limiting the effectiveness of measures adopted for containment. The spread beyond the hospital was later reflected by cases detected through emergency services or with onset in the community, although most cases of community acquisition showed previous contact with healthcare institutions (data not shown).

Patients from whom isolates were recovered had a median age of 70.5 years (range 34–96 years); sex distribution was similar (17 [56.7%] male and 13 [44.3%] female). Median time from admission to positive *bla*_NDM-14_ culture was 14 days (range 0–309 days) (Appendix 2 Figure 1). Most (n = 28) cases were in Canary Islands residents, despite the presence of 2 nonresidents (1 from Morocco, 1 from Germany) in the initial 3-case cluster in ward 10S. Most cases were colonization (19/30 [63.3%]), and 30-day mortality among patients with positive cultures was low (2/30 [6.7%]). With the exception of the index case-patient, who was treated with colistin, most patients with documented infection were treated with ceftazidime/avibactam combined with aztreonam (Appendix 1 Table 1).

### Genomic Investigation and Cross-Border Emergence Linked to Morocco and France

**Figure 2:**
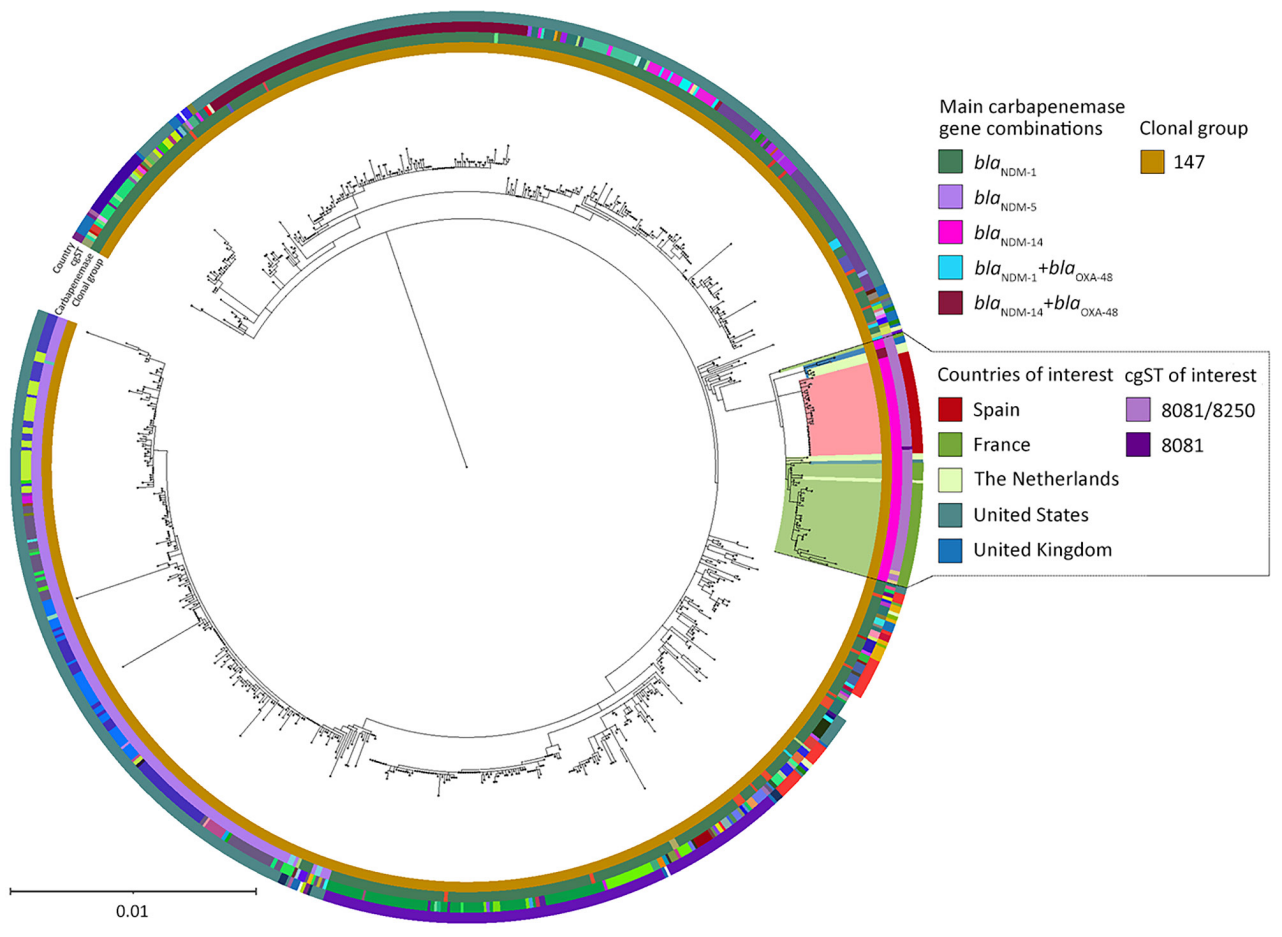
Core-genome phylogenomic tree of New Delhi metallo-β-lactamase (NDM) 14–producing *Klebsiella pneumoniae* ST147 isolates from a Canary Islands outbreak and related isolates from study of emergence of NDM-14–producing *K. pneumoniae* ST147 clone in Spain and outbreak in the Canary Islands. The tree includes the closest 863 genomes of *K. pneumoniae* isolates belonging to the ST147 clone, found in PasteurMLST (https://bigsdb.pasteur.fr/klebsiella), the National Center for Biotechnology Information Genome Database (https://www.ncbi.nlm.nih.gov/genome), and Pathogenwatch (https://pathogen.watch). Red shading indicates the Canary Island outbreak isolates; other countries and STs of interest are also highlighted. The most prevalent carbapenemases appearing in the dataset of the most similar isolates are noted. *K. pneumoniae* isolate TGH13 (GenBank accession no. CP012745.1) was used as the reference strain. Scale bar indicates nucleotide substitutions per site. cgST, core-genome ST; ST, sequence type.

We performed core-genome phylogenomic reconstruction of the 30 NDM-14–producing *K. pneumoniae* isolates identified at CHUIMI in comparison with the closest 863 genomes of the ST147 available in multiple databases and using the genome of *K. pneumoniae* isolate TGH13 (GenBank accession number CP012745.1) as reference. The NDM-14–producing ST147 *K. pneumoniae* isolates from the Canary Islands grouped into a cluster belonging to core-genome multilocus sequence type (MLST) 8081/8250 (Figure 2), which included other genetically related *bla*_NDM-14_–positive ST147 *K. pneumoniae* isolates from other countries, such as France, the Netherlands, the United States, and the United Kingdom. Isolates from the Canary Islands formed a well-defined subcluster, highlighting their close phylogenomic relationship.

We detected 3 core-genome clusters of 5 single-nucleotide polymorphisms (SNPs) among the isolates (Appendix 2 Figure 2). The Canary Islands sublineage had an average core-genome distance among any 2 isolates of 2.7 SNPs and a maximum distance of 9 SNPs. Finally, the mean (+SD) whole-genome distance of any Canary Islands isolate against the index case, I238, was 4 (± 3) mutations (including insertions and deletions). To visualize the strain evolution, we sequenced 3 additional isolates from 2024 (n = 1) and 2025 (n = 2) (Appendix 1 Table 2); those isolates showed an average whole-genome distance of 28 (± 13) mutations against I238.

### Virulome, Resistome, and Plasmidome of NDM-14–Producing *K. pneumoniae* ST147 Isolates from the Canary Islands

Whole-genome sequencing analysis of the 30 isolates identified in 2023 (I238-I440) confirmed clonal dissemination of this ST147 clone (core-genome MLST ≈8081/8250; wzi64, O1/O2v1). Putative virulence was influenced by the presence of aerobactin (*iuc*) and yersiniabactin (*ybt*) and the absence of colibactin (*clb*). The multidrug-resistant profile was associated with the presence of multiple β-lactamase-encoding genes, including *bla*_NDM-14_, *bla*_CTX-M-15_, *bla*_OXA-1_, *bla*_OXA-9_, *bla*_SHV-11_, and *bla*_TEM-1_, as well as detection of a truncated *ompK35* (R60fs), impairing outer membrane permeability. Additional resistance determinants were identified for aminoglycosides (*aac(6′)-Ib′*, *aac(6′)-Ib-cr*, *aadA*, *aph(3′)-VI*, *aph3-Ia*, *armA*), fluoroquinolones (*qnrS1*), and other antimicrobial families (*sul1*, *sul2*, *dfrA5*, *catB3*, *arr-3*, *mphA*, *mphE*, *mrx*, *msrE*). Furthermore, all isolates carried 6 plasmids: incompatiability group (Inc) FIB+IncHI1B (≈330 kbp), IncFIB (≈1,12 kbp), IncFIB (54 kbp), IncR (≈40 kbp), Col(pHAD28) (4 kbp), and ColRNAI (≈9 kbp). Of note, the *bla*_NDM-14_ gene was located on the nonconjugative IncFIB plasmid of ≈54 kbp. We compiled comparative data regarding the differential genomic features of the isolates from the Canary Islands cluster with other nearby isolates (Figure 2; Appendix 2 Figure 2).

### Genetic Context of β-Lactamases

**Figure 3:**
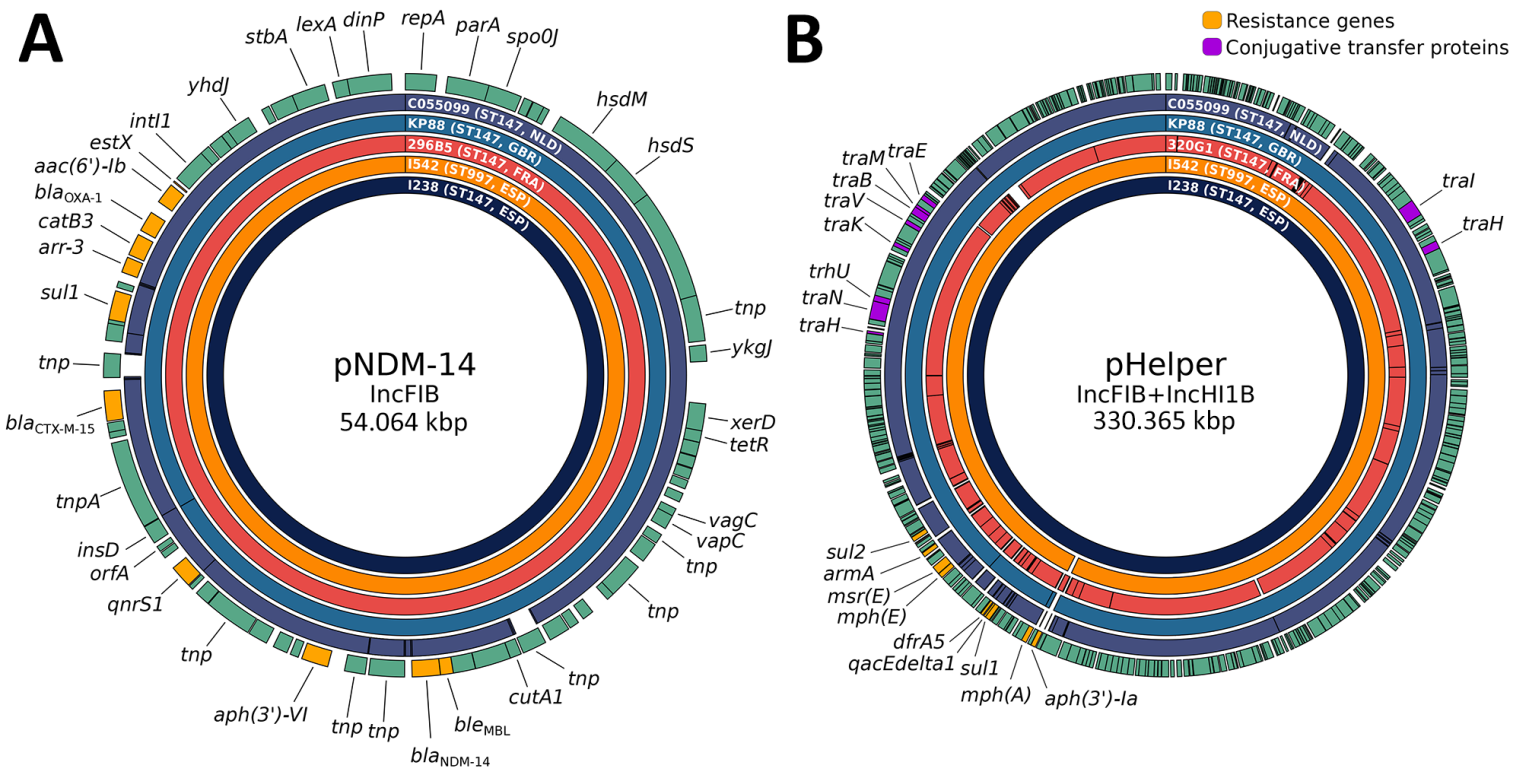
Comparison of 2 of the most relevant plasmids in isolates from study of emergence of New Delhi metallo-β-lactamase (NDM) 14–producing *Klebsiella pneumoniae* ST147 clone in Spain and outbreak in the Canary Islands. The *bla*_NDM-14_–containing plasmid pNDM-14 (A) and its probable helper for conjugation and intraspecies transmission pHelper (B) are shown, comparing multiple *K. pneumoniae* ST147 strains from different countries (Spain, I238; France, 296B5 and 320G1; United Kingdom, KP88; the Netherlands, C055099) and a phylogenomically distant *K. pneumoniae* ST997 carrying the same plasmids (I542, Spain). The outer ring shows loci, including resistance genes and conjugative transfer proteins. Because of the large size of pHelper, only those 2 types of features are shown. The other rings represent the presence or absence of parts of the plasmid relative to the I238 reference plasmid. ESP, Spain; FRA, France; GBR, United Kingdom; Inc, incompatibility group; NLD, the Netherlands; ST, sequence type.

**Figure 4:**
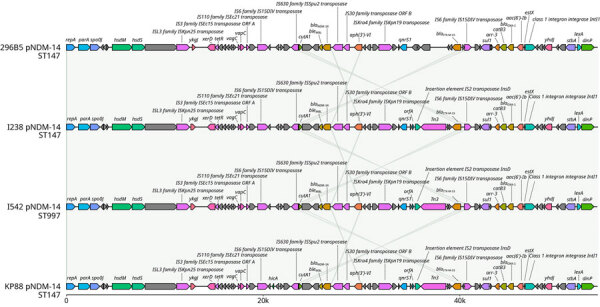
Detailed comparison of circularized *bla*_NDM-14_ plasmids from study of emergence of New Delhi metallo-β-lactamase (NDM) 14–producing *Klebsiella pneumoniae* ST147 clone in Spain and outbreak in the Canary Islands. Plasmids are from multiple countries (Spain, I238 and I542; France, 296B5; United Kingdom, KP88) and 2 different STs. Gray lines indicate synteny at the nucleotide level. Gray shading indicates loci without gene names or named hypothetical proteins; other colors are randomized on the basis of gene order. *ble*_MBL_, bleomycin resistance gene; CTX, cholera toxin; IS, insertion sequence; ORF, open reading frame; OXA, oxacillin; pNDM-14, NDM-14–producing plasmid; ST, sequence type.

The *bla*_NDM-14_ gene was embedded in an IncFIB (54 kbp) plasmid also carrying *bla*_CTX-M-15_ and *bla*_OXA-1_. Another large IncFIB+IncHI1B (≈330 kbp) plasmid harbored multiple resistance genes but also multiple conjugative transfer proteins not present in the IncFIB (54 kbp) plasmid. Of particular interest, an additional isolate, designated I542, which corresponded to a *bla*_NDM-14_–positive *K. pneumoniae* sample detected during the outbreak but belonging to a phylogenomically distant sequence type (ST997), also carried the same plasmids, which confirmed the ability of the *bla*_NDM-14_ gene to spread horizontally among different *K. pneumoniae* sequence types. We further analyzed the IncFIB+IncHI1B (≈330 kbp) and IncFIB (54 kbp) plasmids and compared them with international isolates and MLSTs (Figure 3), finding that they matched almost perfectly with each other. Further analysis of the pNDM-14 plasmid (Figure 4) showed complete synteny at the nucleotide level. The *bla*_NDM-14_ gene was surrounded by an S630 family ISSpu2 transposase upstream and an IS6 family IS15DIV transposase downstream.

### Antimicrobial Susceptibility to β-Lactams and β-Lactam/β-Lactamase Inhibitor Combinations and Mechanisms of Cefepime/Taniborbactam Resistance 

Among the 30 *K. pneumoniae* clinical isolates associated with the outbreak, the most active antimicrobial combinations were aztreonam/avibactam and aztreonam/nacubactam, both demonstrating activity against the whole collection (100% susceptibility rate; 30/30 isolates). Aztreonam/avibactam showed the greatest potency; 50% MIC and 90% MIC values were both 0.125 mg/L. Combinations of cefepime with the novel diazabicyclooctane β-lactamase inhibitors zidebactam and nacubactam also exhibited high levels of activity; susceptibility rates were 96.7% (29/30 isolates), and MIC ranges were 1–16 mg/L for zidebactam and 2 to >32 mg/L for nacubactam. Cefiderocol and colistin both demonstrated similarly high susceptibility rates (96.7% susceptibility rate; 29/30 isolates); cefiderocol displayed a MIC range of 1–4 mg/L and a 50%/90% MIC of 2/2 mg/L, at the clinical breakpoint for susceptibility.

By contrast, all isolates were resistant to piperacillin/tazobactam, ceftazidime, ceftazidime/avibactam, cefepime, aztreonam, imipenem, imipenem/relebactam, meropenem, meropenem/vaborbactam, tobramycin, and amikacin (Appendix 1 Table 4). Of note, the combination of cefepime and the boronate-type β-lactamase inhibitor taniborbactam showed limited activity, inhibiting only 10% of isolates (3/30), with MIC values ranging from 4 to 32 mg/L.

We also evaluated production of NDM-1 or NDM-14 β-lactamases in either the wild-type *E. coli* TG1 host or the porin-deficient *E. coli* HB4 host (Appendix 1 Table 5). The susceptibility profile remained largely unchanged between hosts, with the notable exception of cefepime/taniborbactam, for which the MIC increased substantially, from 4 mg/L in *E. coli* TG1 to 64 mg/L in *E. coli* HB4.

## Discussion

RELAP (*19*) was launched in 2022, aligning with the European Centre for Disease Prevention and Control roadmap for integrating genomic typing in surveillance and outbreak investigations (*22*). The network provides timely, high-resolution WGS-based data on pathogen emergence within the archipelago, with particular focus on high-priority carbapenem-resistant Enterobacterales designated by the World Health Organization.

Using that network, RELAP, located in Tenerife (Spain), was able to carry out WGS-guided monitoring of the emergence of a large outbreak caused by a highly resistant NDM-14–producing *K. pneumoniae* ST147 clone in the Canary Islands. The outbreak affected 99 patients and spread rapidly across 4 hospitals on 3 islands in 27 months. Whereas the ST147 *K. pneumoniae* clone is a known disseminator of NDM-type carbapenemases (*23*,*24*), its association with the NDM-14 enzyme had not previously been reported in Spain. Our findings are consistent with and expand upon a previous report of this clone’s involvement in multiple outbreaks across France (*18*). Direct epidemiologic links with Morocco were detected in both the Canary Islands and France outbreaks.

The Canary Islands’ insular geography makes them highly vulnerable to pathogen importation through intense cross-border mobility (e.g., tourism, medical transfers), whereas their compartmentalized structure contributes to concentrated early transmission within a single island. This effect is further amplified by a tightly interconnected healthcare circuit relying on few reference hospitals, but interisland mobility eventually enables dissemination to other islands. Consequently, whereas the focal nosocomial cluster in this outbreak was controlled, the lineage appears to have disseminated across the community and various islands through patient movement and interisland healthcare referral, no longer limited to a single transmission chain or hospital ward. Therefore, the situation seems to have evolved from a focal outbreak to a scenario of cryptic dissemination. Those aspects highlight the necessity for robust surveillance measures to limit CPE spread in island settings, reinforcing the importance of surveillance networks using high-resolution, WGS-guided methods for rapid outbreak typing and control.

Comparative genomic analysis of the first 30 cases of the outbreak confirmed the NDM-14 enzyme was not acquired by existing *K. pneumoniae* ST147 lineages circulating in Spain, where this clone has been present for more than a decade (*25*,*26*). None of the outbreak isolates were clustered with previously reported ST147 genomes from Spain, suggesting a recent introduction of this specific sublineage of the clone into the country. All isolates from the Canary Island outbreak (average core-genome SNP distance of 2.7) fell within the clonality range (*27*). We also found them to be closely related to NDM-14–producing ST147 *K. pneumoniae* isolates from France, further supporting the hypothesis of a common origin in North Africa, where this high-risk clone was reported in 2025 (*28*). Its presence in the Netherlands, the United States, and the United Kingdom further underscores its global spread. Recent public health communications have argued for improved surveillance of NDM-producing isolates in patients transferred from Ukraine (*29*,*30*). In this context, the ongoing outbreaks in France and the Canary Islands, both linked to patients transferred from Morocco, also illustrate the need to maintain and reinforce surveillance on patient transfers from neighboring North Africa regions with a high burden of CPEs, such as Morocco (*28*), Tunisia (*31*), and Egypt (*32*).

The Canary Islands isolates’ resistome showed remarkable similarity to other NDM-14–producing *K. pneumoniae* isolates publicly available, including carriage of *bla*_NDM-14_, *bla*_CTX-M-15_, *bla*_OXA-1_, *bla*_OXA-9_, *bla*_SHV-11_, and *bla*_TEM-1_. All those genes are also present in genomes found in France, the Netherlands, the United States, and the United Kingdom, highlighting the still limited evolution of the β-lactam resistance mechanisms in this expanding strain. Alarmingly, some recent isolates from the Canary Islands appear to have also acquired *bla*_OXA-48_, as observed in the Netherlands. Another worrying finding is the first identification of the NDM-14–bearing plasmid outside of the ST147 clone in a phylogenomically distant *K. pneumoniae* ST997 isolate from the Canary Islands. Of note, that isolate not only contained the *bla*_NDM-14_ IncFIB (54 kbp) plasmid, which lacks putative genes involved in mobilization, but also the pHelper plasmid previously found to be necessary for transmission. Those observations confirm previous laboratory conjugation experiments (*18*) and demonstrate that conjugation can also occur between clinical strains, highlighting that this gene might disseminate further. Currently, its interspecies distribution seems limited to *Acinetobacter lwoffii* and *K. pneumoniae*. A very similar *bla*_NDM-1_–carrying ≈54 kbp IncFIB plasmid has been seen in Enterobacterales, primarily in *K. pneumoniae* in multiple STs globally. Nonetheless, the NDM-14 variant has only been detected in isolates epidemiologically associated with the North Africa region. That fact, combined with the distinct clustering of the *K. pneumoniae* lineage in which it appears, strongly suggests that the *bla*_NDM-14_–carrying plasmid evolved independently within the North Africa region from a common *bla*_NDM-1_ ancestor.

NDM-producing Enterobacterales are commonly resistant to all classic β-lactams, including aztreonam because of simultaneous production of AmpCs or extended-spectrum β-lactamases. As expected, all our isolates fit this assumption. Cefiderocol was active against the first 30 outbreak isolates analyzed here, although in all cases, MICs were borderline at 2 mg/L. The use of cefiderocol against NDM-producing Enterobacterales is to some extent concerning, because of measurable hydrolysis of this cephalosporin at the biochemical level (*33*) but also because of previously reported cases of drug resistance. By contrast, aztreonam/avibactam was highly active (MICs <0.125–0.25 mg/L), as previously reported (*18*), confirming that combination as the therapeutic option of choice for combating infections caused by the strain. We also analyzed the performance of some developmental combinations in clinical trial evaluation (cefepime/taniborbactam [*34*,*35*], cefepime/zidebactam [*36*], cefepime/nacubactam [*37*], and aztreonam/nacubactam [*37*]) with potential activity against MBL-producing Enterobacterales. However, none of those options was more active than aztreonam/avibactam, because none of them yielded MICs <1 mg/L, thus reinforcing the prominent role that aztreonam/avibactam is expected to have against NDM-producing Enterobacterales in the future (*38*). However, of concern is that some of the newly developed agents, particularly cefepime/taniborbactam, for which phase 3 clinical trial evaluations have recently been completed (*39*), were not active against many of the isolates included here (considering a clinical breakpoint of 4 mg/L). Resistance to that combination might not be caused by direct NDM-mediated resistance to taniborbactam inhibition but instead by a combination of NDM production with loss-of-function mutations affecting the *ompK35* porin channel, as previously observed (*40*). Parallel production of both NDM-1 and NDM-14 in wild-type (*E. coli* TG1) and porin-deficient (*E. coli* HB4) hosts confirmed that NDM production under low-permeability conditions markedly increases cefepime MICs (from 64–128 to >256 mg/L) and cefepime/taniborbactam MICs (from 4 to 64 mg/L), providing further evidence on the interplay between NDM production and reduced outer membrane permeability in cefepime/taniborbactam resistance.

In conclusion, we report a large outbreak and the rapid, widespread emergence of NDM-14–producing *K. pneumoniae* ST147 in the Canary Islands of Spain during 2023–2025. The outbreak began after the direct transfer of a patient from Morocco. This clone is genetically closely related to isolates currently expanding in France and other countries, thus highlighting its clonal cross-border dissemination. The study findings also demonstrate that the *bla*_NDM-14_–carrying plasmid can be efficiently transferred to distantly related *K. pneumoniae* lineages and investigational β-lactam/β-lactamase inhibitor combinations show limited activity against these strains, for which aztreonam/avibactam is likely to become a key therapeutic option in severe infections.

Appendix 1 Additional data for emergence of New Delhi metallo-β-lactamase 14–producing *Klebsiella pneumoniae* sequence type 147 clone in Spain and outbreak in the Canary Islands.

Appendix 2 Additional information for emergence of New Delhi metallo-β-lactamase 14–producing *Klebsiella pneumoniae* sequence type 147 clone in Spain and outbreak in the Canary Islands.
