# Supplementary material for: Emergence of New Delhi Metallo-β-Lactamase 14–Producing Klebsiella pneumoniae Sequence Type 147 Clone in Spain and Outbreak in the Canary Islands
Source: Emerg Infect Dis. 2026 Jan;32(1):63–73. doi: 10.3201/eid3201.251504 (PMC12870054; doi:10.3201/eid3201.251504)
Supplement: Appendix 2 — Additional information for emergence of New Delhi metallo-β-lactamase 14–producing Klebsiella pneumoniae sequence type 147 clone in Spain and outbreak in the Canary Islands. [file 25-1504-Techapp-s2.pdf]

Article DOI: https://doi.org/10.3201/eid3201.251504

EID cannot ensure accessibility for supplementary materials supplied by authors. Readers who have difficulty accessing supplementary content should contact the authors for assistance.

Emergence of New Delhi Metallo-β-Lactamase 14–Producing *Klebsiella pneumoniae* Sequence Type 147 Clone in Spain and Outbreak in the Canary Islands

Appendix 2

Supplementary methods

Screening of carbapenemase production

Colonization by carbapenemase-producing isolates at CHUIMI was screened using the CHROMID® Carba agar (bioMérieux, France) selective and differential chromogenic medium. Presumptive carbapenemase-producing isolates were further checked for carbapenemase production using the RAPIDEC® CarbaNP colorimetric test (bioMérieux, France), as well as the Rosco KPC/metallo-β-lactamase and OXA-48 confirm kit. Carriage of NDM-type carbapenemase genes was genetically confirmed using a MDR Direct Flow CHIP (Vitro diagnostica, Spain).

Bioinformatic analysis

Illumina short reads were quality controlled with fastp (v.0.23.2, https://github.com/OpenGene/fastp), long PacBio reads were quality controlled with filtlong (v.0.2.1, https://github.com/rrwick/Filtlong) and host contamination was removed using BMTagger (v.3.101, https://ftp.ncbi.nlm.nih.gov/pub/agarwala/bmtagger). Assemblies were created through Unicycler (v.0.5.0, https://github.com/rrwick/Unicycler), utilizing a hybrid approach when possible, and polished with Polypolish (v.0.5.0, https://github.com/rrwick/Polypolish). They were then assessed for contamination and completeness with CheckM (v.1.1.3, https://github.com/Ecogenomics/CheckM). Raw reads and assemblies are available in BioProject PRJNA1216752, with their complete accession IDs

(Biosample, SRA, Genome) available in Appendix 1 Table 2 (https://wwwnc.cdc.gov/EID/article/32/1/25-1504-App1.xlsx). Multilocus sequence type (MLST) and core genome MLST (cgMLST) were identified with Pathogenwatch (v.23.1.5, https://pathogen.watch). Antibiotic resistance genes were detected through RGI (v.5.2.0, https://github.com/arpcard/rgi) and CARD (v.3.2.8, https://card.mcmaster.ca). Plasmids were identified using MOB-suite (v.3.1.0, https://github.com/phac-nml/mob-suite). For samples where plasmids were not properly detected by this tool, additional confirmation was performed through read alignment with bwa (v.0.7.18) [https://github.com/lh3/bwa] when possible, declaring a plasmid present if the coverage and depth of the expected plasmid was confidently high. Kleborate (v.3.1.0, https://github.com/klebgenomics/Kleborate) was additionally used for virulence and antibiotic resistance characterization. Core genome phylogenomic analysis was performed using snippy (v.4.6.0, https://github.com/tseemann/snippy), with Klebsiella pneumoniae TGH13 (CP012745.1, ST147) as reference and a total of 2353 ST147 isolates from multiple databases like PasteurMLST (https://bigsdb.pasteur.fr/klebsiella), NCBI (https://www.ncbi.nlm.nih.gov) and Pathogenwatch up to December, 2024. The closest 863 isolates to our samples were then selected (Appendix 1 Table 3) to create a circular phylogenomic tree using fasttree (v.2.1.11, https://morgannprice.github.io/fasttree, with parameters "-nt -gtr") and iTOL (v.7, https://itol.embl.de). Moreover, the 46 closest isolates were again selected in order to make a zoomed-in view with ggtree (v.3.0.4, https://github.com/YuLab-SMU/ggtree). Plasmid comparison visualizations were created through Brick (v.f5aa1b9, https://brick.ink/) and gggenomes (v.1.0.1, https://github.com/thackl/gggenomes).

Susceptibility testing

Antimicrobial susceptibility testing of piperacillin/tazobactam, ceftazidime/avibactam, cefepime, aztreonam, imipenem, imipenem/relebactam, meropenem, meropenem/vaborbactam, tobramycin, amikacin and colistin was determined using Sensititre EUMDRXXF microdilution plates (ThermoFisher Scientific, Dardilly, France). Minimum inhibitory concentrations (MICs) of cefepime, cefepime/taniborbactam, cefepime/zidebactam, cefepime/nacubactam, aztreonam, aztreonam/avibactam, aztreonam/nacubactam, and cefiderocol, were determined in triplicate by reference broth microdilution assays with cation-adjusted Müeller-Hinton (MH) broth, and for cefiderocol, iron-depleted cation-adjusted MH broth prepared according to CLSI M100

guidelines (https://clsi.org/standards/products/microbiology/documents/m100). MIC values were interpreted following EUCAST guidelines (v. 15.0) for Enterobacterales (www.eucast.org/clinical_breakpoints). Clinical breakpoints for combinations that have not yet been approved were interpreted using the β-lactam partner. Avibactam and taniborbactam were tested at a fixed concentration of 4 mg/L, while zidebactam and nacubactam was fixed at a 1:1 ratio. Reference strains *Escherichia coli* ATCC 25922, *Klebsiella pneumoniae* ATCC 700603, *Escherichia coli* NCTC 13353 and *Acinetobacter baumannii* NCTC 13304 (for cephalosporin/β-lactamase inhibitor combinations) and *K. pneumoniae* ATCC BAA-2814 (for carbapenem/β-lactamase inhibitor combinations) were used as controls. Minimum inhibitory concentrations (MICs) are shown in Appendix 1 Table 4.

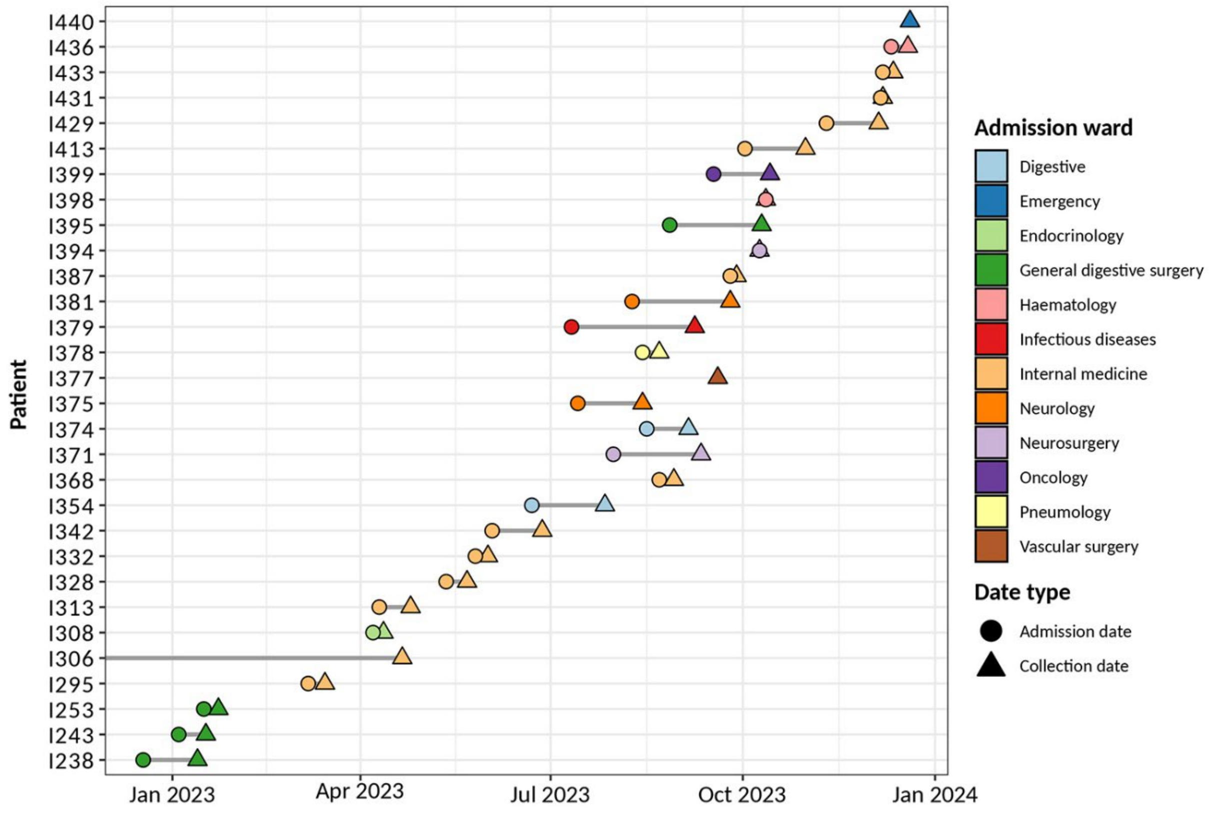

Appendix 2 Figure 1. Timeline of NDM-14-producing *Klebsiella pneumoniae* positive cultures, showing patient admission date and corresponding dates of positive samples during a tertiary care hospital outbreak in Gran Canaria, Spain, 2023.

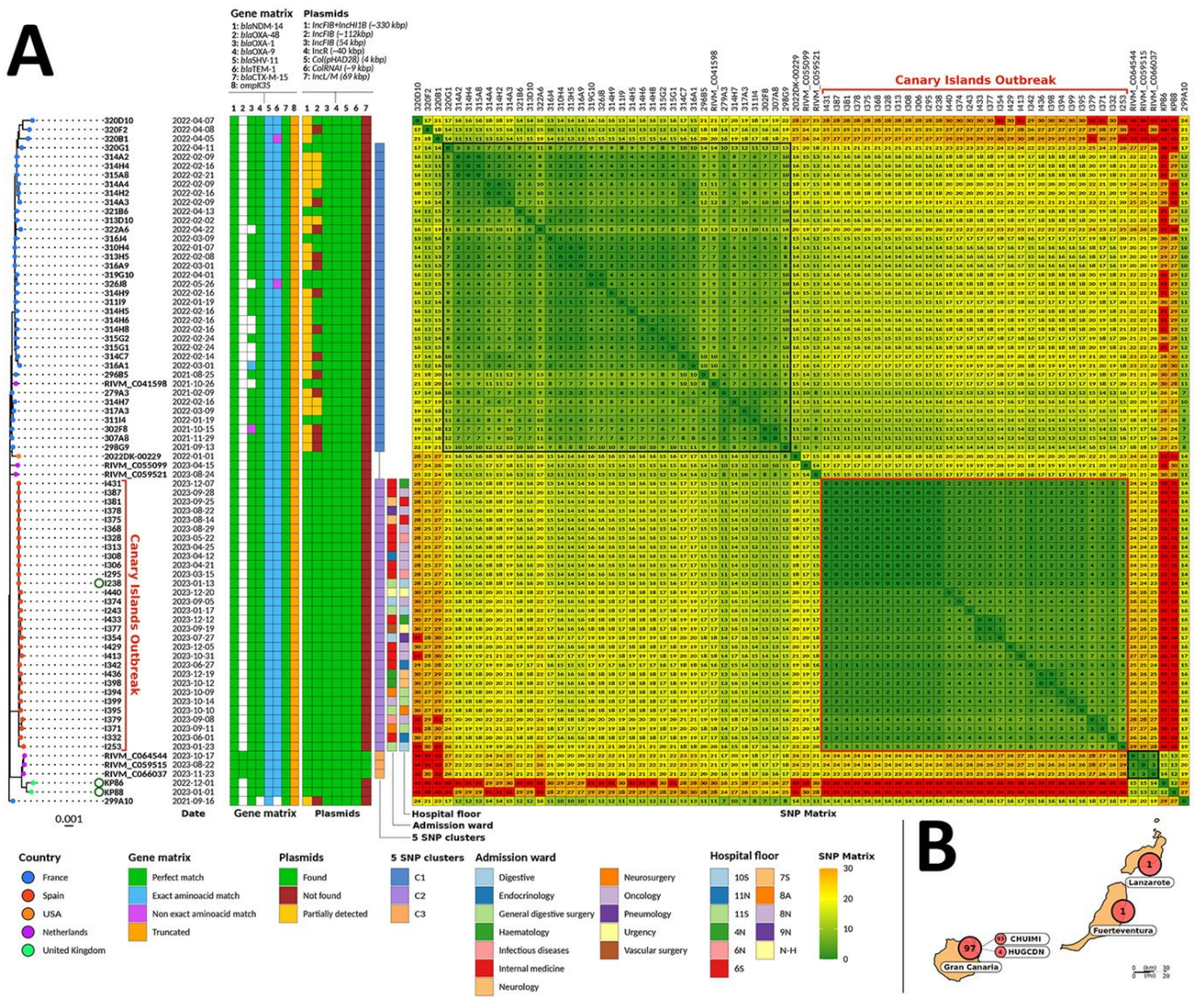

Appendix 2 Figure 2. Core genome phylogenomic tree of the closest 46 *K. pneumoniae* ST147 to the initial 30-case Canary Islands outbreak in 2023 (A) and Isolate geographic distribution in the Canary Islands between 2023 and 2025 (B) from study of emergence of New Delhi metallo-β-lactamase 14–producing *Klebsiella pneumoniae* sequence type 147 clone in Spain and outbreak in the Canary Islands. A) Countries, date of first positive sample, β-lactamases, plasmid content, core genome SNP clusters, admission ward, hospital floor information related to the outbreak and core genome Single Nucleotide Polymorphism (SNP) matrix are shown. Additionally, complete genomes are indicated by a green circle next to their names. *Klebsiella pneumoniae* TGH13 (CP012745.1, ST147) was used as the reference strain. N-H: non-hospitalized. B) Outbreak map displaying the geographic distribution of all 99 confirmed cases detected between 2023 and 2025 across the affected portion of the Canary Islands.
